# An Overview of Bioactive Phenolic Molecules and Antioxidant Properties of Beer: Emerging Trends

**DOI:** 10.3390/molecules28073221

**Published:** 2023-04-04

**Authors:** Mirella Nardini

**Affiliations:** CREA, Research Centre for Food and Nutrition, Via Ardeatina 546, 00178 Rome, Italy; mirella.nardini@crea.gov.it

**Keywords:** beer, polyphenols, antioxidant activity, adjunct, fruit, vegetables, herbs, natural food

## Abstract

Beer is one of the oldest and most common beverages worldwide. The phenolic contents and antioxidant properties of beer are crucial factors in evaluating its nutritional quality. Special beers brewed with the addition of adjuncts are gaining in consumer preference, in response to demands for healthy food and new gustatory and olfactory stimuli. Many studies recently dealt with functional beers brewed with the addition of adjuncts. This review focuses on bioactive molecules, particularly the composition of phenolic compounds, and the antioxidant activity of beer. The current knowledge concerning the effect of the addition of adjuncts in the form of fruit, vegetables, herbs, and natural foods on the polyphenol content, antioxidant properties, and phenolic profile of beer is reviewed, with an outline of the emerging trends in brewing processes. Future studies need to complete the identification and characterization of the bioactive molecules in beer, as well as studying their absorption and metabolic fate in humans.

## 1. Introduction

Oxidative stress is a process in which the physiological balance between pro-oxidants and antioxidants is disrupted, resulting in potential damage to human health [1]. Dietary antioxidants may represent a crucial strategy in counteracting the negative effects of oxidative stress.

Diets rich in fruits and vegetables are associated with healthy effects, such as reduced risk of chronic diseases and cancer. Polyphenols constitute the most abundant class of natural dietary antioxidants, being present in virtually all fruits and vegetables. They may act as reducing agents, free-radical scavengers, singlet oxygen quenchers, and, potentially, as chelators of prooxidants. Epidemiological studies strongly indicated that the long-term consumption of polyphenol-rich foods exerts protection against the development of cardiovascular disease, cancer, diabetes, neurodegenerative diseases, and ageing [2,3,4,5,6,7].

Depending on diet composition and individual habits, polyphenol intake may be of several hundred milligrams per day, largely exceeding that of other dietary antioxidants, such as vitamin E, vitamin C, and carotenes [8,9]. For individuals regularly consuming wine, coffee, beer, fruit juice, chocolate, and tea, these beverages will be the major sources of phenolic antioxidants [9,10]. The study of the health effects of alcoholic beverages has largely focused on wine. Several epidemiological studies reported significant reductions in all-cause and, particularly, cardiovascular mortality in moderate wine drinkers compared to abstainers and to individuals drinking excess alcohol [11,12,13]. Moderate beer consumption has also been associated with a decreased incidence of cardiovascular disease, hypertension, diabetes, neurodegenerative disease, cancer, and osteoporosis [14,15,16,17,18,19]. Beer drinking has been reported to increase plasma antioxidant capacity and anticoagulant activities, to positively affect plasma-lipid levels in humans, and to alleviate post-menopausal symptoms in women [20,21,22].

Beer is a popular product consumed in large amounts all over the world as nutritious and refreshing beverage. Beer is a source of carbohydrates, amino acids, minerals, vitamins, and bioactive compounds, such as polyphenols, particularly phenolic acids, in the form of benzoic and cinnamic-acid derivatives and flavonoids. The bioactive compounds in beer naturally result from its ingredients or are produced through the brewing process (secondary metabolites from yeast and lactic acid bacteria). It has been reported that about 30% of beer polyphenols originate from hops, and the remaining 70% from malt [23,24,25]. Moreover, polyphenols may be metabolized during the brewing process, which may enhance or reduce their potential activity [26].

The quality of beer may be improved through novel brewing approaches, in terms of ingredients, brewing method, and fermentation type, to increase its content of bioactive compounds and to lower its alcohol content. The growing demand for a great variety of beer types from consumers, including alcohol-free beers, has led producers to explore and increase the range of ingredients, as well as renewing interest in different yeasts and fermenting bacteria. In addition to the most familiar products, beers produced by the addition of fruit, spices, vegetables, and natural foods during the fermentation process are becoming very popular all over the world, responding to demands for new gustatory, olfactory, and visual stimuli from consumers. The addition of whole fruit to beer is traditionally practiced in Belgium to produce cherry lambic (Kriek) or raspberry lambic (Framboise) by adding sour cherries (*Prunus cerasus* L.) or raspberries (*Rubus idaeus* L.) to fermenting lambic beer. The sugar present in fruit triggers a secondary fermentation. During the refermentation and maturation of fruit beers, flavors and bioactive compounds, particularly carotenoids and polyphenols (the latter being quantitatively the most abundant), are extracted from the fruit.

In this review, attention is focused on bioactive molecules, particularly phenolic compounds, and the antioxidant activity of beer. The novelty of this review is that the phenolic composition of the main beer ingredients (hops, barley, wheat, rye, and oats) is reviewed and quantitative data are presented for each phenolic molecule. Moreover, the effects the addition of fruit, vegetables, herbs, and natural foods (adjuncts) on the polyphenol content and antioxidant properties of beer, in comparison with conventional beers, are reviewed, outlining the emerging trends in the brewing process. As a further novelty, the identification and quantitation of single phenolic molecules in beer brewed with or without adjuncts are reviewed and presented for all the classes of phenolic molecules under study.

## 2. Methodology

A systematic literature search through PubMed, Scopus, and Web of Science databases was carried out. The original articles investigating the antioxidant properties and bioactive phenolic molecules of beer were identified using the following search keywords: polyphenols and beer, flavonoids and beer, fruit and beer, health and beer, spice and beer, special beer, flavoring beer, hop, rye, oat, barley, wheat, vegetables, lignan, stilbenes, tyrosol, hydroxytyrosol, alkylresorcinols. A large amount of research (range, 1992–2022) was selected according to the relevance to the topic. The focus was on the new insights regarding bioactive phenolic molecules and antioxidant properties of beer obtained with or without adjunct addition during brewing process. A few studies (4) in which the amount of added adjunct/L beer was not specified were not included in this review.

The CAS (Chemical Abstract Service) numbers of the phenolic compounds are: caffeic acid, 331-39-5; chlorogenic acid, 327-97-9; *p*-coumaric acid, 501-98-4; ferulic acid, 1135-24-6; gallic acid, 149-91-7; gentisic acid, 490-79-9; *p*-hydroxybenzoic acid, 99-96-7; protocathecuic acid, 99-50-3; syringic acid, 530-57-4; *o*-coumaric acid, 614-60-8; 2,4-dihydroxybenzoic acid, 89-86-1; sinapic acid, 530-59-6; vanillic acid, 121-34-6; 2,6-dihydroxybenzoic acid, 303-07-1; homovanillic acid, 306-08-1; 4-hydroxyphenylacetic acid, 156-38-7; salycilic acid, 69-72-7; catechin, 154-23-4; epigallocatechin, 970-74-1; epicatechin, 490-46-0; isorhamnetin, 480-19-3; kaempferol, 520-18-3; naringenin, 480-41-1; naringin, 10236-47-2; procyanidin B1, 20315-25-7; procyanidin B2, 29106-49-8; procyanidin C1, 37064-30-5; quercetin, 117-39-5; rutin, 153-18-4; genistein, 446-72-0; formononetin, 485-72-3; luteolin, 491-70-3; hesperidin, 520-26-3; myricetin, 529-44-2; apigenin, 520-36-5; malvidin 643-84-5; vicenin II, 23666-13-9; vitexin, 3681-93-4; daidzein, 486-66-8; avenanthramide A, 108605-70-5; avenanthramide B, 108605-69-2; avenanthramide C, 116764-15-9; desmethylxanthohumol, 115063-39-3; isoxanthohumol, 70872-29-6; 8-prenylnaringenin, 68682-02-0; xanthohumol, 6754-58-1; *trans*-resveratrol, 501-36-0; *trans*-piceid, 27208-80-6; *cis*-piceid, 148766-36-3; *cis*-resveratrol, 61434-67-1; tyrosol, 501-94-0; hydroxytyrosol, 10597-60-1; delphinidin galactoside, 28500-00-7; cyanidin galactoside, 142506-26-1; cyanidin rutinoside, 28338-59-2; pelargonidin galactoside, 34425-22-4; pelargonidin rutinoside, 33978-17-5; quercetin glucuronide, 22688-79-5; kaempferol galactoside, 23627-87-4; kaempferol 3-*O*-rutinoside, 31921-42-3.

## 3. Bioactive Compounds from Common Ingredients

Beer can be limited to four ingredients: barley, hops, water, and yeast. The addition of yeast was introduced late, once its role in the fermentation process was understood. In addition to barley, different cereal grains and carbohydrate sources may be used, such as wheat, rye, and oats. In Table 1, the main bioactive phenolic compounds found in hops, barley, wheat, oats, and rye are reported. Concerning the phenolic acid content, the data in the literature are not homogeneous. Many authors report the levels of the free form of phenolic acids, while some others refer to the contents of total, conjugated, or bound phenolic acids. Free phenolic acids make the smallest contribution to the total phenolic acid content. In cereals, the free-phenolic-acid contribution to the total phenolic acid level has been reported to be typically <0.5–1.0%, while soluble conjugated and bound phenolic acids represent the greatest proportion of the total phenolic acid content [27].

In this review, unless otherwise specified, the concentration of total/conjugated phenolic acids is reported. When this is not possible due to a lack of data from the literature, the concentrations of the free forms of phenolic acids are reported and specified.

**Hops**, (*Humulus lupulus* L.), an angiosperm plant (Cannabaceae family), initially used for their preservative properties, became appreciated over the years for the bitterness and aroma they impart to beer. Hops are largely used in traditional medicine. The potentially healthy effects of hops have been attributed to their polyphenols, which exert antioxidant activities and enhance nitric-oxide production [28]. About 1000 different polyphenolic substances have been found in hop cones, accounting for about three-to-eight percent of dry hop cones. The positive healthy effects of hop polyphenols on various chronic diseases, such as menopause [29], cancer [30,31,32,33], inflammation [31,32,34], arthritis [32], insulin sensitivity, type II diabetes, and metabolic syndrome [35] have been described. Further, hops have been reported to contain antimicrobial compounds, associated with a group of 56 phenolic compounds, with a wide range of potential activity, including the inhibition of virus replication and bacterial and fungal growth [36]. The polyphenol content of hops may vary greatly due to cultivation conditions, the time of harvest, and further modifications occurring during processing, brewing, and storage [28,37,38]. Hop bracts have been described as containing 40–140 mg/g polyphenols [33]. The phenolic composition of hop extracts has been reported to be more than 55% proanthocyanidins and more than 28% flavonoid glycosides [33].

The phenolic contents of hops are reported in Table 1. Caffeic, chlorogenic, *p*-coumaric, gallic, syringic, protocatechuic, ferulic, *p*-hydroxybenzoic, and gentisic phenolic acids have been identified [28,39,40,41]. Among flavonoids, the flavonols isorhamnetine, kaempferol, quercetin, catechin, epigallocatechin, epicatechin, and the flavanones naringenin and naringin have been measured by liquid chromatography–mass spectrometry (LC-MS) [28,41]. The glycosylated flavonoid rutin was also detected [39]. Moreover, the oligomeric flavonoid proanthocyanidins and the prenylflavonoids xanthohumol, isoxanthohumol, desmethylxanthohumol, and 8-prenylnaringenin have been identified [28,35,39,40]. Xanthohumol may represent about 1% of the dry weight of hop cones [42]; however, due to its hydrophobicity, its content in wort and beer is low. Xanthohumol becomes isomerized to isoxanthohumol during hop boiling [35]. Furthermore, 8-prenylnaringenin, has been reported to possess high estrogenic activity [42]. In addition, other biological activities have been ascribed to prenylflavonoids, including the inhibition of bone resorption and osteoporosis prevention [43,44], the inhibition of the cytochrome-P450-mediated activation of procarcinogens [45,46], antiproliferative effects on breast- and colon-cancer cell lines [46], and anti-angiogenic activity [47,48].

The stilbene *trans*-resveratrol and its glucoside *trans*-piceid were detected in American and European hops by means of high-performance liquid chromatography (HPLC-MS) [25,49]. Their content varies greatly in the range of 0.5–11.7 mg/kg (Table 1). This discovery opens new perspectives for understanding the health benefits of hops. In fact, the cardioprotective effects of red wine have been attributed to resveratrol [50]. These effects include antiplatelet, anti-inflammatory, estrogenic, cardioprotective, antitumor, and antiviral properties [50,51]. The resveratrol glucoside *trans*-piceid seems to possess very similar biological activity [51,52].

A large proportion of beer phenolics derives from **barley** (*Hordeum vulgare* L.). These are especially present as phenolic acids, flavonoids, lignans, and alkylresorcinols (Table 1). The main phenolic acids in barley are the protocatechuic, *p*-hydroxybenzoic, 2,4-dihydroxybenzoic, chlorogenic, gallic, vanillic, syringic, ferulic, caffeic acid, *p*-coumaric, *o*-coumaric, and sinapic acids [53,54,55,56,57,58,59,60]. Among flavonoids, flavan-3-ols (catechin, epicatechin), flavonols (kaempferol, myricetin, quercetin, rutin), flavones (apigenin, tricin, saponarin), flavanones (naringenin, naringin, hesperidin), proanthocyanidins, and anthocyanins (cyanidin malonyl glucoside, cyanidin 3-galactoside, cyanidin 3-glucoside, pelargonidin, peonidin 3-glucoside, cyanidin acetyl galactoside, delphinidin 3-glucoside, malvidin 3-glucoside) have been identified [55,58,59,61]. The most abundant polyphenols in barley have been reported to be ferulic acid and procyanidin B [62].

Alkylresorcinols, also known as resorcinolic lipids, are bioactive phenolics composed of long aliphatic chains and resorcinol-type phenolic rings. They are present in whole-grain cereals, such as wheat, barley, and rye. The total content of alkylresorcinols in barley is reported in Table 1. It varies in the ranges of 3.2–10.3 mg/100 g DW, according to Andersson et al. [56], and 28.6–35.4 mg/100 g FW, according to Mattila et al. [53]. In vivo and in vitro studies and epidemiological investigations showed that ARs can affect many physiological and pathological processes related to the immune system, metabolic regulation, cell signaling, and gene expression. They have been shown to exhibit antimicrobial, anticancer, antilipidemic, and antioxidant activities [63,64,65].

Lignans are natural polyphenols commonly present in plants. Barley has been reported to contain lignans (total 1.25 mg/100 g FW, Table 1) [60]. Furthermore, HPLC/MS analyses led to the identification of pinoresinol, medioresinol, syringaresinol, lariciresinol, cyclolariciresinol, secoisolariciresinol, secoisolariciresinol-sesquilignan, matairesinol, 7-oxomatairesinol, 7-hydroxymatairesinol, todolactol A, and α-conidendrin, with pinoresinol, lariciresinol, 7-hydroxymatairesinol, and syringaresinol as the major lignan components [60]. Due to their structural similarity to estradiol, lignans are classified as phytoestrogens. Lignans have been shown to be converted by the intestinal microflora to the mammalian lignans enterodiol and enterolactone in human intervention studies [60,66]. The effects on human health and diseases of phytoestrogens, including lignans, and their metabolites have been widely reviewed, including their effects on cancer and cardiovascular diseases [67,68,69,70]. Lignans have been described as possessing a variety of biological properties, such as antioxidative, antitumor, antiviral, antibacterial, antifungal, estrogenic, and cardioprotective activities [67].

In addition to polyphenols, barley also contains tocopherols (alpha, gamma, and delta), tocotrienols, carotenoids (lutein and zeaxanthin), and phytosterols [56,71]. All these phytochemicals exhibit strong antioxidant, antiproliferative, and cholesterol-lowering activities, which are potentially useful in lowering the risk of certain human diseases, such as cancer, cardiovascular disease, diabetes, and obesity [72]. Malt, which is used for the manufacturing of beer, contains various compounds from barley, such as phenolic compounds, and from the malting process, such as Maillard-reaction products, which can play significant roles in malting and brewing through their antioxidant properties [73,74,75]. Regarding the malting process, the contents of some phenolic compounds and their antioxidant activities have been reported to increase remarkably during the later stages of germination and subsequent kilning [76]. Malt extracts from barley have been reported to contain high amounts of polyphenols, to scavenge hydroxyl and superoxide radicals, and to confer protection against reactive-oxygen-species-induced lipid, protein, and DNA damage [74].

Another common ingredient of beer is **wheat** (*Triticum aestivum* L.), which is used in large amounts to produce wheat beers. Wheat, as with barley and all cereals, is an important source of dietary polyphenols. Compared with vegetables and fruits, the contents and biological activities of polyphenols in cereals have long been underestimated. The contribution of cereals to the intake of dietary polyphenols in Spain has been reported to be around 360 mg/day [16]. The chief phenolics in wheat are phenolic acids, flavonoids, alkylresorcinols, and lignans (Table 1). Among phenolic acids, ferulic, gallic, vanillic, caffeic, *p*-coumaric, *p*-hydroxybenzoic, sinapic, and syringic acids have been detected, with ferulic acid the most abundant. The flavonoids catechin, epicatechin, apigenin, malvidin, and luteolin glucosides have been detected, with catechin and apigenin as the most abundant [16,54,63,77]. Proanthocyanidins (procyanidins B3, prodelphinidin, and propelargonidin) have also been identified in wheat [61].

Alkylresorcinols and lignans are also present in wheat (Table 1) [27,53,54,77,78]. Alkylresorcinols are present in wheat at high levels, in the range of 19.1–142.9 mg/100 g DW [27,53,54,77,78,79,80]. The total lignans have been reported to be 9.22 mg/100 g FW, with hydroxymatairesinol, secoisolariciresinol, lariciresinol, siringaresinol, iso-hydroxymatairesinol, and 7-oxo-matairesinol as the major lignans. In addition, the stilbene pinosylvin has been identified in wheat [60,81].

In addition to wheat, the use of oats and rye in beer production is also frequent [82,83,84]. In addition to being good sources of carbohydrates, **oats** (*Avena sativa* L.) contain a range of phenolic antioxidants. The main polyphenols in oats are avenanthramides, phenolic acids, and flavonoids (Table 1). Among phenolic acids, gallic, protocathecuic, *p*-hydroxybenzoic, caffeic, syringic, *p*-coumaric, ferulic, sinapic, and vanillic acids have been detected, with ferulic acid being the most abundant [53,85,86]. Furthermore, the flavonoids vicenin II, vitexin, daidzein, and apigenin glucoside arabinoside have been identified [77].

Avenanthramides are phenolic alkaloids consisting of amide conjugates of anthranilic acid (an aromatic β-amino acid) or 5-hydroxyanthranilic acid, with hydroxycinnamic acids, mainly *p*-coumaric acid (avenanthramide A), ferulic acid (avenanthramide B), and caffeic acid (avenanthramide C). They have been reported to possess antioxidant activities, with caffeic and ferulic acid derivatives being the most effective compounds [87]. The avenanthramide content of oats is reported in Table 1 [53,77,85,86,88,89]. Avenanthramide B is the most abundant, followed by avenanthramide A and avenanthramide C. Remarkably, avenanthramides that are unique to oats have been reported to be bioavailable in humans and may exert anti-inflammatory, antiproliferative, antiatherogenic, and vasodilatory effects, as well as protecting against cardiovascular diseases and colon cancer [90,91,92,93,94].

The rate of lignans in oats were reported to be 3.49 mg/100 g FW by Smeds et al., with pinoresinol, lariciresinol, 7-hydroxymatairesinol, matairesinol, and syringaresinol as major components [60].

**Rye** (*Secale cereale* L.) is a traditional dietary ingredient in some regions, especially Northern and Western Europe. It is an important source of bioactive compounds (Table 1). Furthermore, it has been reported to contain polyphenols, alkylresorcinols, and lignans [66]. The major antioxidant phenolic compounds in rye are the following phenolic acids (103–300 mg/100 g DW): *p*-hydroxybenzoic, vanillic, syringic, ferulic, *p*-coumaric, caffeic, and sinapic acids. Of these, ferulic acid is the most abundant [66,95,96]. Among flavonoids, the rye flavones crysoeriol, apigenin, and tricin were identified in the form of glycosides [97,98]. The alkylresorcinol contents of rye are reported in Table 1. Alkylresorcinols are of particular interest due to their potential use as biomarkers of rye and wheat intake [53,66,80,96]. In fact, among the cereals used for human food, alkylresorcinols are present at high levels only in rye and wheat. Purified alkylresorcinols from rye have been reported to be incorporated into human erythrocyte membranes and to protect erythrocyte-membrane lipids from peroxide-induced oxidation [99].

Among rye lignans, syringaresinol, pinoresinol, lariciresinol, secoisolariciresinol, matairesinol, isolariciresinol, 7-hydroxymatairesinol, 7-oxo-matairesinol, cyclolariciresinol, and medioresinol have been identified, with lariciresinol, pinoresinol, and syringaresinol as the major lignans [60,66].

**Table 1:** Contents of bioactive phenolic compounds in common beer ingredients.

	Phenolic Compound	Reference
**Hops**	***Phenolic acids* ^a^:**	
	*Caffeic acid*: 0.01–15.8 mg/100 g DW	[28,39,41]
	*Chlorogenic acid*: 0.47–163.7 mg/100 g DW	[28,39,41]
	*p-Coumaric acid*: 0.01–28.8 mg/100 g DW	[28,39]
	*Ferulic acid*: 0.01–0.10 mg/g DW	[39]
	*Gallic acid*: 0.08–3.41 mg/g DW	[39,41]
	*Gentisic acid*: 1.5–6.7 mg/100 g DW	[28]
	*p*-*Hydroxybenzoic acid*: 1.87 ± 0.01 mg/g DW	[41]
	*Protocatechuic acid*: 0.42–2.25 mg/g DW	[39,41]
	*Syringic acid*: 0.03–12.9 mg/g DW	[28,39,41]
	***Flavonoids*:**	
	*Catechin*: 1.2–56.1 mg/100 g DW	[28,39,41]
	*Epigallocatechin*: 10.3–28.6 mg/100 g DW	[28]
	*Epicatechin*: 0.08–8.4 mg/100 g DW	[28,39]
	*Isorhamnetin*: 0.5–3.3 mg/100 g DW	[28]
	*Kaempferol*: 0.44–49.4 mg/100 g DW	[28,41]
	*Naringenin*: 3.9–11.0 mg/100 g DW	[28]
	*Naringin*: 1.7–3.9 mg/100 g DW	[28]
	*Procyanidin B1:* 18.4–50.6 mg/g DW	[28]
	*Procyanidin B2*: 8.4–14.6 mg/g DW	[28]
	*Procyanidin C1*: 3.8–16.9 mg/g DW	[28]
	*Quercetin*: 1.03–111.8 mg/100 g DW	[28,41]
	*Rutin*: 0.61–0.88 mg/g DW	[39]
	***Prenylflavonoids*:**	[48]
	*Desmethylxanthohumol*: 120.0 mg/100 g DW	[28,48]
	*Isoxanthohumol*: 8.0–35.2 mg/100 g DW	[28,48]
	*8-Prenylnaringenin*: 1.5–23.8 mg/100 g DW	[28,39,40,48]
	*Xanthohumol*: 85.6–480.0 mg/100 g DW	
	***Stilbenes*:**	[25,49]
	*Total trans-Stilbenes:* 0.05–1.17 mg/100 g FW	[25]
	*trans-Resveratrol*: 0.003–0.228 mg/100 g FW	[25]
	*trans-Piceid*: 0.04–1.10 mg/100 g FW	
**Barley**	***Phenolic acids*:**	
	*Total*: 0.2–67.5 mg/100 g DW; 16.5–24.1 mg/100 g FW	[53,56,59]
	*Caffeic*: 0.17 ± 0.01 mg/100 g FW	[53]
	*Chlorogenic*: 0–9.84 mg/100 g DW	[58]
	*o*-*Coumaric*: 1.5–6.0 mg/100 g DW	[58]
	*p*-*Coumaric*: 4.0 ± 0.49 mg/100 g FW; 0.17–58.3 mg/100 g DW	[53,56,58]
	*Gallic*: 0.1–136.6 mg/100 g DW	[58]
	*p*-*Hydroxybenzoic*: 0.31 ± 0.05 mg/100 g FW; 0.58–2.67 mg/100 g DW	[53,56]
	*2,4-Dihydroxybenzoic*: 0.68–6.16 mg/100 g DW	[56]
	*Protocatechuic*: 0.16 ± 0.01 mg/100 g FW	[53]
	*Sinapic acid*: 1.10 ± 0.17 mg/100 g FW; 0.14–2.44 mg/100 g DW	[53,56]
	*Syringic*: 0.50 ± 0.03 mg/100 g FW; 0.1–91.6 mg/100 g DW	[53,56,58]
	*Ferulic*: 25.0 ± 3.2 mg/100 g FW; 0.59–4.25 mg/100g DW	[53,56,58]
	*Vanillic acid*: 0.71 ± 0.08 mg/100 g FW; 0.10–3.91 mg/100 g DW	[53,56,58]
	***Flavonoids*:**	
	*Total*: 6.2–30.1 mg/100 g DW; 7.8–16.2 mg/100 g FW	[57,59]
	*Catechin*: 0.1–10.5 mg/100 g DW; 0.48–2.4 mg/100 g FW	[57,58,59,100]
	*Hesperidin*: 0.5–24.9 mg/100 g DW	[58]
	*Kaempferol*: 1.27–19.2 mg/100 g DW; 1.2–2.4 mg/100 g FW	[57,58,59]
	*Myricetin*: 0–73.3 mg/100 g DW; 3.1–4.3 mg/100 g FW	[57,58,59]
	*Naringin*: 0.77–6.97 mg/100 g DW	[58]
	*Naringenin*: 4.7–50.2 mg/100 g DW	[58]
	*Quercetin*: 2.0–8.7 mg/100 g DW; 1.5–6.7 mg/100 g FW	[57,58,59]
	*Rutin*: 1.4–11.8 mg/100 g DW; 0.07–0.46 mg/100 g FW	[58,59]
	***Alkylresorcinols*:** 3.2–10.3 mg/100 g DW; 2.86–3.54 mg/100 g FW	[53,56,80]
	***Lignans*: *Total*:** 1.25 mg/100 g FW	[60]
***Wheat***	***Phenolic acids*:**	
	*Caffeic*: 0–3.3 mg/100 g DW; 98.6 ± 11.9 mg/100 g FW	[27,78]
	*p*-*Coumaric*: 0.30–1.21 mg/100g DW; 0.38–3.7 mg/100 g FW	[27,53]
	*Ferulic*: 10.0–219.3 mg/100 g FW; 0.94–6.23 mg/100 g DW	[27,53,78]
	*Gallic acid*: 2.8 ± 0.4 mg/100 g FW	[78]
	*p*-*Hydroxybenzoic*: 0.23–1.11 mg/100 g DW	[27]
	*Sinapic*: 2.8–12.8 mg/100 g DW; 0.8–6.3 mg/100 g FW	[27,53,78]
	*Syringic*: 0.39–2.22 mg/100 g DW; 0.22–1.3 mg/100 g FW	[27,53]
	*Vanillic*: 0.88–2.45 mg/100 g DW; 0.37–17.6 mg/100 g FW	[27,53,78]
	***Flavonoids*:**	
	*Apigenin*: 20.4 ± 1.5 mg/100 g FW	[78]
	*Catechin*: 94.2 ± 5.6 mg/100 g FW	[78]
	*Epicatechin*: 14.5 ± 0.9 mg/100 g FW	[78]
	*Malvidin*: 2.9 ± 0.2 mg/100 g FW	[78]
	***Alkylresorcinols*:**	
	*Total*: 19.1–142.9 mg/100 g DW; 74.8–76.62 mg/100 g FW	[53,79,80]
	*Lignans*:	
	*Total*: 9.22 mg/100 g FW	[60]
**Oats**	***Phenolic acids*:**	
	*Total:* 35.1–143.5 mg/100 g DW; 46.9–65.1 mg/100 g FW	[53,86,89]
	*Caffeic*: 0.95–7.02 mg/100 g DW; 0.11–1.94 mg/100 g FW	[53,85,89]
	*p*-*Coumaric*: 1.3–2.2 mg/100 g DW; 0.21–1.2 mg/100 g FW	[53,85,86]
	*Ferulic*: 4.5–19.0 mg/100 g DW; 2.3–33.0 mg/100 g FW	[53,85,86,89]
	*Gallic acid*: 14.4–70.4 mg/100 g DW	[89]
	*p*-*Hydroxybenzoic*: 3.2–6.0 mg/100 g DW; 0.33–2.2 mg/100 g FW	[53,85,86]
	*Protocathecuic acid*: 1.1–10.4 mg/100 g DW	[89]
	*Sinapic*: 3.4–5.2 mg/100g DW; 0.9–3.6 mg/100 g FW	[53,85,86]
	*Syringic*: 2.5–5.0 mg/100 g DW; 0.68–2.8 mg/100 g FW	[53,85,86]
	*Vanillic*: 1.8–42.7 mg/100 g DW; 0.4–2.4 mg/100 g FW	[53,85,86,89]
	***Flavonoids*:**	
	*Vicenin II*: 0.70 ± 0.02 mg/100 g DW	[77]
	*Vitexin*: 2.47 ± 0.19 mg/100 g DW	[77]
	*Daidzein*: 2.92 ± 0.01 mg/100 g DW	[77]
	*Apigenin-6/8-C-pentoside-8/6C-hexoside I*: 1.15 ± 0.84 mg/100 g DW	[77]
	***Avenanthramides*:**	
	*Total*: 4.2–14.7 mg/100 g DW; 1.3–5.0 mg/100 g FW	[53,77,85,86,88,89]
	*Avenanthramide A*: 0.68–2.38 mg/100 g FW	[85]
	*Avenanthramide B*: 1.2–3.7 mg7100 g FW	[85]
	*Avenanthramide C*: 0.1–1.24 mg/100 g FW	[85]
	***Lignans*:**	
	*Total:* 3.5 mg/100 g FW	[60]
**Rye**	***Phenolic acids*:**	
	*Total:* 49.1–300.0 mg/ 100 g DW	[66,96]
	*Caffeic*: 0.4–7.7 mg/100 g DW	[66]
	*p*-*Coumaric*: 0.74–6.5 mg/100 g DW	[95,96]
	*Ferulic*: 3.5–117.4 mg/100 g DW	[95,96]
	*p*-*Hydroxybenzoic*: 0.7–2.4 mg/100 g DW	[66]
	*Sinapic*: 5.2–14.0 mg/100 g DW	[95,96]
	*Syringic*: 0.02–0.6 mg/100 g DW	[66,96]
	*Vanillic*: 0.46–4.6 mg/100g DW	[66,96]
	***Flavonoids*:**	
	*Total:* 4.2–20.4 mg/100 g DW	[98]
	Total flavones: 5.6–13.7 mg/100 g DW	[97]
	***Alkylresorcinols*:** 36–320 mg/100 g DW; 2–130 mg/100 g FW	[53,66,80,96]
	***Lignans*:**	
	*Total:* 0.11–2.27 mg/100 g DW; 11.2 mg/100 g FW	[60,66]

Values reported represent concentration range or mean ± standard deviation. FW = fresh weight; DW: dry weight. ^a^ For hops, the contents of free forms of phenolic acids are reported.

## 4. Polyphenol Contents and Antioxidant Properties of Conventional Beers

Several studies investigated the characteristics of beers with respect to their phenolic contents and to their antioxidant properties. The total polyphenol content of beer is conventionally measured by the colorimetric Folin assay. For conventional beers, the total polyphenols content has been reported to range from 88 mg/L to 500 mg/L of gallic acid equivalents (GAE) for the most common beer types [20,84,101,102,103,104,105,106]. The content greatly depends on the beer type, with highest values measured in abbey, bock, and black beers, (622, 875 and 855 mg/L GAE respectively) [104,105,106]. A total polyphenol content of 1366 mg/L GAE has occasionally been reported for a Danish porter beer [107]. The total flavonoid content is usually measured by the aluminum chloride colorimetric method. For conventional beers, it has been reported to vary from 26.6 mg/L to 73.2 mg/L of catechin equivalents (CATE) [84,103,108].

Regarding the antioxidant activities of conventional beers, these have been measured by several assays, most frequently ABTS, FRAP, and DPPH. The antioxidant activity measured by the ABTS method gave values in the range of 0.16–2.74 mM Trolox equivalents (TE) for conventional beers [84,101,102,103,107,109,110]. The results reported in the literature for FRAP measurements commonly vary from 1.50 to 4.66 Fe_2_SO_4_ mM equivalents, with the higher values measured in ale, bock, and abbey [84,103,104,111]. For FRAP measurements reported as mM TE instead of Fe_2_SO_4_, the values are in the range 0.51–2.56 mM TE [107,109,112,113]. A value of 4.12 mM TE has occasionally been reported for a Danish porter beer [107]. The antioxidant activity of beer evaluated with the DPPH method gives values in the range of 0.24–1.35 mM TE for conventional beers [101,102,110,111,113], whereas higher values have been described for some craft beers (1.44 mM TE/L; 4.81 mM TE/L; 2.27 mM TE/L [109,112,114]).

A significant correlation has been reported between the total polyphenol content and antioxidant activity of beer [84,101,102,103,104]. Similarly, a strict correlation was found between the total flavonoid content and antioxidant activity of beer [84,103,104].

## 5. Identification of Bioactive Phenolic Molecules in Beers

In addition to the evaluation of the total polyphenol and flavonoid contents and to the measurement of antioxidant activity, many studies focused on the identification of bioactive phenolic molecules in beers. The total polyphenol and flavonoid contents were measured with colorimetric methods, while for the characterization and identification of bioactive phenolic molecules, high-resolution techniques were employed, including high-performance liquid chromatography (HPLC), high-performance liquid chromatography coupled with mass spectrometry (HPLC-MS), ultra-high-performance liquid chromatography (UHPLC), ultra-high-performance liquid chromatography coupled with mass spectrometry (UHPLC-MS), nuclear magnetic resonance (NMR), gas chromatography (GC), and gas chromatography coupled with mass spectrometry (GC-MS). Table 2 summarizes the phenolic molecules identified in conventional beers. The main phenolic compounds in beer are phenolic acids in the form of benzoic- and cinnamic-acid derivatives. Ferulic acid, caffeic acid, vanillic acid, homovanillic acid, sinapic acid, *p*-coumaric acid, *o*-coumaric acid, gallic acid, protocatechuic acid, 4-hydroxyphenylacetic acid, gentisic acid, chlorogenic acid, *m*-coumaric acid, 2,6-dihydroxybenzoic acid, *m*-hydroxybenzoic acid, *p*-hydroxybenzoic acid, salicylic acid, and syringic acid have been identified in conventional beers, with ferulic acid as the most abundant phenolic acid, followed by caffeic, sinapic, *p*-coumaric, and vanillic acids [84,101,103,104,115,116,117,118]. The results in Table 2 show that most phenolic acids are present in beer in conjugated, esterified forms, while free forms are present at lower levels. Most of the antioxidant activity of beer (55–88%) has been reported to be derived from six phenolics, with ferulic acid representing over 50%, followed by syringic acid, catechin, caffeic acid, protocatechuic acid, and epicatechin [101]. A strict correlation has been reported between antioxidant activity and the total phenolic acid contents of beer, particularly ferulic, protocathecuic, caffeic, syringic, sinapic, *p*-coumaric, 4-hydroxy-phenylacetic, and vanillic acids [101,103,104]. Phenolic acids exhibit strong antioxidant properties and have been reported to play a role in ameliorating ischemia/reperfusion injury, inflammation, Alzheimer’s and Parkinson’s disease, diabetes mellitus, and skin disease [119,120,121,122]. They have also been described as possessing antidepressant-like effects [123] and anticancer activity [124,125,126,127].

Among flavonoids, catechin, epicatechin, rutin, quercetin, naringenin, apigenin, luteolin, and kaempferol have been identified in beers (Table 2) [101,105,118,130,131]. Catechin, epicatechin, and rutin have been detected in some commercial beers. Rutin, quercetin, and kaempferol have also been identified in detectable amounts in one out of three ale beers by HPLC-ESI-MS [129]. Very low amounts of the flavonoids genistein, biochanin A, daidzein, and formononetin (<0.004 mg/L) have been detected in conventional beers by radioimmunoassay [134]. A significant correlation was found between antioxidant activity and total flavonoids content [84,103]. Among flavonoids, a strict correlation has been reported between the antioxidant activity and the catechin and epicatechin contents of beer [100]. However, it must be taken into consideration that flavonoids in beers have been reported as present at very low levels in many studies, and are often undetectable [84,103,112,132,134,135].

The prenylflavonoid contents in beers are shown in Table 2. Isoxanthohumol, xanthohumol, and 6-prenylnaringenin, which are the most abundant compounds, have been reported to constitute more than 90% of the total amount of prenylflavonoids in beer, with 8-prenylnaringenin and 6-geranylnaringenin being minor components [136,137,138,139]. The content of 8-prenylnaringenin has been reported to be higher in ale and stout beers (0.011–0.021 mg/L) compared to lager beers (0–0.0089 mg/L), due to the generally rich hopping of ales (“triple” beers) [136,137,138,139,140]. However, it has been reported that many beers do not contain prenylflavonoids in detectable amounts [137,139,141]. Prenylflavonoids are present at high levels in hops, with their content varying across hop varieties [28,39,40,48]. They are transferred to beer during brewing, although, due to their hydrophobicity, their content in beer is low.

The contents of the stilbene *trans*-resveratrol and its glycoside *cis*-piceid in beer have been reported to be in the ranges of 0–0.067 and 0–0.024 mg/L, respectively, with lower levels of *cis*-resveratrol and *trans*-piceid (Table 2). Resveratrol was found in 79% out of 110 beers analyzed, while piceid was present in only 33% of them [142]. These stilbene derivatives have been found in some hop varieties, which are used for beer production and are transferred to beer during brewing [25]. Regarding the stilbene resveratrol, bioavailability studies on humans demonstrated its absorption and rapid metabolism; the glucuronide and sulfate conjugates were found to be the major plasma and urine metabolites [3]. Resveratrol has been reported to have beneficial effects on cardiovascular disease, aging, Alzheimer’s disease, cancer, and numerous health-promoting properties, such as antioxidant, anti-inflammatory, anti-diabetes, anti-obesity, and antiproliferative effects, in both animals and humans [143,144,145].

The presence of the phenolic alcohols tyrosol and hydroxytyrosol has been reported in beer, ranging from 0.2 to 44.4 mg/L and from 0.0 to 0.13 mg/L, respectively (Table 2) [117,136,146]. Tyrosol, hydroxytyrosol, and their metabolites exhibited strong antioxidant activities and antiatherogenic, cardioprotective, anticancer, neuroprotective, and endocrine effects in in vivo and in vitro studies [147,148,149,150,151]. Hydroxytyrosol has been demonstrated to be absorbed from the diet in humans and to be present in plasma and urine in conjugated forms [151,152,153]. Moreover, one clinical trial showed that tyrosol is absorbed from beer in humans and endogenously bio-transformed into hydroxytyrosol [146].

Alkylresorcinols have also been identified in beer, in the range of 0.02–11.04 µg/L beer (Table 2) [136]. Alkylresorcinols are present at high levels in barley and wheat and are transferred to beer during the brewing process. Similarly to prenylflavonoids, the low content of alkylresorcinols in beer may be due to their hydrophobicity.

## 6. Polyphenol Contents and Antioxidant Properties of Beers with Added Fruit, Vegetable, Herbs, and Natural Food

Recently, characterizations of the bioactive phenolic compounds and antioxidant activity in several fruit beers have been reported [84,103,109,110,111,112,114,135,154,155,156,157,158,159]. Fruit beers obtained through the addition of fresh fruits (hairy-fig fruits, *Ficus hirta* Vahl. [154]; persimmon fruits, *Diospyros kaki* L [155]; quinces, *Cydonia oblonga* Miller [156]; peaches, *Prunus persica* L. [103,157]; apricots, *Prunus armeniaca* L. [103]; grapes, *Vitis vinifera* L. [103,158,159]; plums, *Prunus domestica* L. [103]; oranges, *Citrus sinensis* L. [103]; apples, *Malus domestica* L. [103]; cherries, *Prunus avium* L. [103,112]; raspberries, *Rubus idaeus* L. [103]; walnuts, *Juglans regia* L. [84]; chestnuts *Castanea sativa* L. [84]; saskatoon-berry fruits, *Amelanchier alnifolia* Nutt. [114]; goji berries, *Lycium barbarum* L. [135]; omija fruits, *Schisandra chinensis* L. [111]; mango, *Mangifera indica* L. [109]; and dotted hawthorn, *Crataegus punctata* L. [110] during the fermentation process resulted in significant enrichment in phenolic compounds, in both quality and quantity, and considerable improvements in antioxidant activities compared to conventional beers. A similar trend has been observed for beers obtained through the addition of vegetables, herbs, and natural foods during the fermentation process: cocoa (*Theobroma cacao* L.) beans [84], honey (*Wildflower honey*) [84], green tea (*Camelia sinensis* L.) [84], coffee (*Coffea arabica* L. and *Coffea robusta* L.) [84], licorice (*Glycyrrhiza glabra* L.) [84], eggplant (*Solanum melongena* L.) peel extract [108], green pepper (*Capsicum annuum* L.) [160], propolis extract [113] and hibiscus (*Hibiscus sabdariffa* L.) [161] extract, green-pepper basil (*Ocinum selloi* benth) [162], *Parastrephia lucida* leaves [107], lemon-balm leaf (*Melissa officinalis* L.) [163], thyme (*Thymus vulgaris* L.) [163], juniper berries (*Juniperus communis* L.) [163], nettle root (*Urtica dioica* L.) [163], hop cones (*Humulus lupulus* L.) [163] and olive leaves (*Olea europaea* L.) [164].

Table 3 summarizes the data from the literature concerning the total polyphenol and flavonoid contents of special beers obtained through the addition of fruits, vegetables, herbs, or natural foods during the brewing process in comparison to control beers obtained without the addition of adjuncts (values in parentheses). The amount of adjuncts added during the brewing process varies from 0.25 to 300 g/L in beer. In most of the studies, the adjuncts added were fresh fruits and vegetables. Goji berries and omija fruits were used in dried forms. In nine studies, the adjunct was added in the form of an extract, particularly water extracts for hibiscus and ethanol/water extracts for propolis, eggplant peel, melissa, thyme, nettle root, juniper berries, hop cones, and urtica root. Beers produced with the addition of fruits, vegetables, herbs, or natural foods have been reported to exhibit total polyphenol contents that are significantly higher than those measured in control beers without the addition of adjuncts, except for apple and apricot beers (Table 3). The polyphenol concentrations were measured in 37 out of the 38 reports. The highest values (>700 mg/L GAE equivalents) were measured in beers with added cherries, chestnuts, cocoa beans, green pepper, hibiscus extract, *Parastrephia lucida* leaves, licorice, and walnuts, regardless of the amount of adjunct added. Similarly, the total flavonoid contents of the beers with added fruits, vegetables, herbs, or natural foods, measured in 19 out of the 38 reports, were reported to be significantly higher compared to those of control beers without adjuncts, except for apple, apricot, green tea, and honey beers (Table 3). Cherry, cocoa-bean, eggplant-peel extract, grape, licorice, omija-fruit, orange-peel, peach, plum, raspberry, and walnut beers exhibited the highest values (>80 mg/L catechin equivalents), regardless of the amount of adjunct added (Table 3).

Regarding the antioxidant activity of special beers obtained with the addition of fruit, vegetables, herbs or natural foods, the antioxidant activity was measured with a FRAP assay in 29 out of the 38 reports reported in Table 3. The strongest antioxidant activity was measured in cherry, chestnut, cocoa-bean, coffee, grape, licorice, orange-peel, peach, plum, raspberry, juniper-berry-extract, Melissa-extract, *Parastrephia lucida*-leaf, thyme-extract, and walnut beers (≥4.5 mM Fe_2_SO_4_ or TE equivalents), regardless of the amount of adjunct added. The antioxidant activities measured in the beers with adjuncts added has been reported to be significantly higher than those measured in the control beers with no addition of adjuncts, except for apple, apricot, green-tea, honey, and saskatoon-berry beers. The antioxidant activity was evaluated with an ABTS assay in 25 out of the 38 reports reported in Table 2. The highest ABTS values were detected in cherry, chestnut, cocoa-bean, coffee, goji-berry, grape, hibiscus-extract, honey, licorice, orange-peel, *Parastrephia lucida*-leaf, hop-cone, juniper-berry-extract, melissa-extract, nettle-root extract, thyme-extract, and walnut (≥2.5 mM TE) beers, regardless of the amount of adjunct added. The antioxidant activities measured with the ABTS assay in beers with added fruits, vegetables, herbs, or natural foods were reported to be significantly higher in than those measured in control beers with no adjuncts, except for apple, apricot, quince, green-tea, peach, plum, and honey beers. A DPPH assay was used in 19 out of the 38 reports to evaluate the antioxidant activities of beers with added fruits, vegetables, herbs, or natural foods (Table 3). The highest values (>2.5 mM TE) were measured in cherry, grape, and saskatoon-berry beers. The DPPH values measured in beers with adjuncts were reported to be significantly higher than those obtained in control beers without adjuncts, except for beers with added dotted hawthorn fruit, peaches, and propolis extract. A significant strict correlation between antioxidant activity and total polyphenol/total flavonoid contents was found in beers with added fruits, vegetables, herbs, and natural foods [84,103,113,163]. A correlation between antioxidant activity and total hydroxycinnamic =-acid content has been reported in beer with added quince fruit [156].

## 7. Identification of Bioactive Compounds in Beers with Added Fruits, Vegetables, Herbs, and Natural Food

Recently, many studies described the composition of bioactive molecules of special beers produced with the addition of fruits, vegetables, herbs, and natural foods. Table 4 summarizes the main results obtained in 23 reports. Regardless of the amount of adjunct added, some conclusions may be drawn. The addition of fruits, vegetables, herbs, and natural foods influences the quality and quantity of polyphenols in beer. The concentration of most phenolic acids is generally increased by the addition of adjuncts, particularly chlorogenic, neochlorogenic, caffeic and *p*-coumaric acids, with the extent of the increment varying for the different adjuncts. Chlorogenic acid was present at the highest level in apricot beer (12.71 mg/L), followed by quince, cherry, and plums beers, while neochlorogenic acid exhibited very high concentrations in plum beer (60.3 mg/L), followed by cherry, apricots, and quince beers. The presence of neochlorogenic acid was not reported in conventional beer, while chlorogenic acid was present in conventional beer at low levels (Table 2). Caffeic acid is remarkably high in plum beer (89.80 mg/L), followed by cherry (54.6 mg/L), apricot (17.83 mg/L), peach (16.3 mg/L), grape (13.41 mg/L), and coffee (9.20 mg/L) beers, while the highest value reported for conventional beer was 6.38 mg/L (Table 3). Furthermore, 3,5-Dicaffeoylquinic acid has been detected in quince beers at levels of 3.0–3.2 mg/L (Table 4). The content of *p*-coumaric acid was remarkably high in cherry beer (62.40 mg/L), followed by plum (12.20 mg/L), grape (7.23 mg/L), goji-berry (up to 7.98 mg/L), orange-peel (4.94 mg/L), chestnut (3.36 mg/L), walnut (4.32 mg/L), and cocoa (3.26 mg/L) beers, while the values reported for the other adjuncts were close to those measured in conventional beers (Table 3). For ferulic acid, the highest values were reported for orange peel (27.87 mg/L) and chestnut (27.55 mg/L) beers (Table 4), while for the remaining adjuncts, the concentrations of ferulic acid reported were in the range of those measured in conventional beers (Table 2). The content of vanillic acid in the beers brewed with adjuncts was almost always close to that reported for conventional beers (Table 2). The highest levels were measured in grape (6.98 mg/L) and peach (6.11 mg/L) beers. A similar trend was observed for syringic acid, with values reported for beers with added adjuncts very close to those measured in conventional beers (Table 2), except for grape beer, which exhibited the highest syringic acid content (2.46 mg/L). The content of sinapic acid reported for beers with added adjuncts was also quite close to that measured in conventional beers (Table 2), except for cherry beer, which exhibited the highest sinapic-acid concentration (7.58 mg/L), and licorice and orange-peel beers, with concentrations somewhat higher than those measured in conventional beers (Table 2).

From the data shown in Table 4, it can be concluded that the addition of fruit to beer is responsible for the highest concentration of phenolic acids compared other types of adjunct. Moreover, a significant strict correlation has been reported between antioxidant activity and total phenolic acid content, as measured by HPLC, as well as between antioxidant activity and the caffeic, vanillic, *p*-coumaric, chlorogenic, and sinapic acid contents of fruit beers [103,156].

Among the flavonoids, the catechin, quercetin, and myricetin contents (Table 4) showed remarkable differences compared to conventional beers (Table 2). The catechin content, reported in 11 out of 23 reports, was higher in all the beers with adjuncts than in the conventional beers, except in two cases (green-tea and cocoa-bean beers). The highest catechin concentration was measured in beers brewed with orange peel (10.87 mg/L), followed by apricot (9.99 mg/L), grape (7.30 mg/L), cherry (7.14 mg/L), and plum beer (6.42 mg/L) (Table 4). The presence of quercetin was reported in 14 out of the 23 reports (Table 4). Its concentration was remarkably higher in walnut (6.55 mg/L), honey (4.67 mg/L), cherry (3.99 mg/L), apricot (3.23 mg/L), raspberry (3.07 mg/L), and licorice (2.63 mg/L) beers than in conventional beers (Table 2). A strict correlation between antioxidant activity and catechin and quercetin content has been reported in fruit beers [103]. Myricetin content is reported in 12 out of 23 reports (Table 4). The concentration of myricetin was notably higher in all the beers analyzed than in conventional beers (Table 2), with the highest values detected in licorice beer (8.82 mg/L), followed by plum (5.31 mg/L), walnut (4.44 mg/L), cherry (1.88 mg/L), peach (1.78 mg/L), green-tea (1.69 mg/L), and raspberry (1.54 mg/L) beers. For conventional beers, only one study is available, giving a myricetin concentration in the range of 0.15–0.16 mg/L (Table 2) [128].

The presence of epicatechin and rutin was reported in six out of the twenty-three reports (Table 4), and their concentrations were in the range measured in conventional beers (Table 2), except for beer brewed with goji berries, which exhibited a rutin concentration in the range of 1.38–23.13 mg/L. Derivatives of the flavonoid kaempferol have been identified in beer with added saskatoon berry (Table 4) [114].

The flavonoid derivatives delphinidin galactoside, cyanidin galactoside, cyanidin rubinoside, pelargonidin galactoside, pelargonidin rubinoside, quercetin glucuronide, and kaempferol galactoside have been identified and quantified in cherry beer (Table 4). The presence of the monomeric anthocyanins delphinidin glucoside, delphinidin rutinoside, delphinidin rutinoside glucoside, cyanidin rutinoside, and petunidin rutinoside has been reported in beer brewed with eggplant peels [108]. Monomeric and polymeric anthocyanins have also been identified in beer brewed with hibiscus extract [157].

The presence of lignans was reported in one report out of the 23. The lignans schisandrin (range 8.96–12.10 mg/L), gomisin A (2.19–3.12 mg/L), and gomisin B (0.65–0.86 mg/L) are present in beer with added omija fruits (Table 4).

Regarding the stilbenes, the presence of resveratrol in beer with added adjuncts was reported by 12 out of the 23 reports, in the range of 0.11–2.24 mg/L beer. The highest resveratrol concentrations were found in beer with added grape (2.24 mg/L) and peaches (1.0 mg/L), followed by apple (0.56 mg/L), chestnut, green-tea, cocoa-bean, walnut, honey, coffee, licorice, raspberry, and apricot beers (Table 4). Interestingly, the levels of resveratrol measured in the beers with adjuncts were significantly higher than those reported for conventional beer (0.001–0.077 mg/L, Table 2). Resveratrol is known to be present at high levels in grape and wine. Recently, apple was suggested to be the secondary dietary source of resveratrols, particularly piceid, in the form of resveratrol glycoside [165,166]. Resveratrol and piceid have also been reported to be present in many vegetable foods, such as peanut butter, cocoa, apricots, walnut, pear, plum, honey, persimmon, celery, orange, lemon, strawberry, mulberry, and many others [23,25,49,166,167,168]. Thus, the high level of resveratrol measured in the beers with adjuncts is likely to have arisen from the different adjuncts added during the brewing process, in addition to that derived from hop. Moreover, although the studies mainly focused on resveratrol and its protective effects, evidence has shown that piceid may be absorbed as resveratrol after it is de-glycosilated in the mammalian small intestine and exerts its biological effects [169,170].

In the beer brewed with olive leaves, the presence of oleuropein (42–73 mg/L) and of the phenolic alcohol 3-hydroxytyrosol (43-75 mg/L) has been reported (Table 4). For comparison, 3-hydroxytyrosol is reported to be present in conventional beer in the range of 0.03–0.1 mg/L (Table 2). Therefore, the addition of olive leaves to beer during the brewing process resulted in enrichment with high amounts of oleuropein and in a significant increase in 3-hydroxytyrosol concentrations compared with conventional beers. Oleuropein is the main polyphenol present in olive leaves and fruits. It is a phytoalexin with a catechol function, giving hydroxytyrosol upon hydrolysis. Oleuropein has been reported to produce strong antioxidant and anti-inflammatory activities, as exerting hepatoprotective, anticancer, antidiabetic, neuroprotective, antiatherogenic and cardioprotective effects [151,171]. It was also shown to possess high levels of antimicrobial and antiviral activity.

No data concerning the presence of prenyl flavonoids or alkylresorcinols in beers with adjuncts are reported in the literature.

## 8. Influence of Brewing Processes on Antioxidant Properties and Polyphenol Contents of Beer

The production of beers rich in antioxidants has caught the attention of the brewing industry. The choice of ingredients is critical to obtain beer with high nutritional value in terms of phenolic antioxidant content. Moreover, beers exhibiting high phenolic contents and high antioxidant activity show improved quality, more stable aroma and flavor, foam stability, and longer shelf life compared to beers with lower phenolic levels and weaker antioxidant properties. The addition of fruits, vegetables, herbs, and natural foods during the brewing process results in a significant increase in polyphenol contents, particularly phenolic acids, flavonoids, and stilbenes, and in antioxidant activity compared with conventional beers. The polyphenol contents and the antioxidant activity of beers with adjuncts have been reported to be comparable to or even higher than those measured in white wines [84]. Moreover, the addition of adjuncts during the brewing process may lead to the enrichment of beer with unusual compounds, or it may increase the concentration of molecules that would otherwise be present in beer at low levels. An example of this is the addition of olive leaves during the brewing process, which leads not only to remarkable increases in 3-hydroxytyrosol contents compared to conventional beers, but also leads to the enrichment of the beer with oleuropein, a bioactive compound that is not present at all in conventional beers. The use of wheat and rye might help to increase the alkylresorcinol content of beer, while the use of oats might enrich beer with avenantramides, a particular class of polyphenols with strong antioxidant activity, only present in oats. In addition to polyphenols, beer ingredients and adjuncts, such as fruit, vegetables, herbs, and natural foods also contain other antioxidants, such as carotenoids, tocopherols, ascorbic acid, and Maillard-reaction products; the latter are generated during malting and wort boiling [71,172]. All these compounds, although present in beer at low levels, might contribute to some extent to the overall antioxidant activity of beer.

The beer content of phenolic-antioxidant compounds is also significantly affected by hops, temperature, and the yeast strain [38]. The hopping method appears to play a significant role in the content of phenolic compounds, with higher polyphenol concentrations observed in the boiling stage hopped beer compared to dry hopping. The optimal time and method for adding hops to beer varies, including adding the hops into the mash prior to boiling, adding them later in the brewing process, usually in the last 2–7 days of fermentation (for dry-hopped beers), or using the method of infusing beers with hop extracts at the point of consumption via a Randall. All these approaches have the potential to vary the amount of bioactive compounds in the final beer [173]. Temperature may also influence the polyphenol composition of final beer. It was reported that the content of polyphenols increased as the fermentation temperature decreased. Gallic, chlorogenic, and ferulic phenolic acids were reported to be present at higher concentrations in beer fermented at 18 °C compared to beer fermented at 12 °C, while tyrosol and vanillic acid were higher in beers fermented at 12 °C [174]. The type of yeast strain has also been reported to influence the phenolic content of final beer [174].

The brewing process itself may influence the content of bioactive compounds in the final beer in two possible ways. First, the production of alcohol and increasing alcohol concentrations might help to dissolve compounds from hops. The second potential effect regards the production of secondary metabolites, especially with prolonged fermentation [173]. Leitao et al. investigated the effect of the various processing steps on both the content and the antioxidant activity of beer phenolic compounds [175]. The evaluation of beer antioxidant activity at different steps of beer processing (brewing, boiling, and fermentation) showed that the total antioxidant activity remained unchanged throughout, while the polyphenol content showed a three-fold increase. Hopping and fermentation were the main causes of this increase. However, the increase in the polyphenolic content measured after fermentation could be attributed to the better extraction of polyphenols due to the presence of ethanol, rather than to a real increase in their content [175]. The analytical method used by the authors (liquid chromatography–antioxidant, LC–AOx) showed that the largest antioxidant contribution was from catechin, caffeic acid, ferulic acid, and sinapic acid, in addition to three unidentified compounds, probably with flavanoid structures, such as polymers of gallocatechin. Other authors determined the phenolic contents during the beer-production process, reporting contrasting results, with the contents of the phenolics remaining constant, increasing, or decreasing. Zhao reported that the polyphenol content increases during malting and mashing but decreases significantly during fermentation and storage [176].

Regarding beer with added fruits, vegetables, herbs, and natural food, the adjuncts were added during wort boiling, fermentation, and maturation, or before packaging. Additions performed during the wort-boiling step result in a more efficient extraction of phenolic compounds from the adjunct compared to when the addition is performed in late stages [177]. Ducruet et al. reported the effect of the addition of goji berries at different stages of the brewing process [135]. The addition of goji berries at any stage during the brewing process resulted in a beer with a significantly higher concentration of bioactive compounds and stronger antioxidant activity than the control beer without any adjuncts; the best result was obtained by adding the goji berries to the wort at the beginning of brewing process, before wort boiling [135]. A similar behavior has been described for the addition of omija fruits [111]. On the other hand, it has been observed that the addition of fresh cherry juice before the beginning of the secondary fermentation stage results in polyphenol contents and antioxidant activity that are significantly higher than those obtained by adding fresh cherry juice to wort before the beginning of the primary fermentation stage [112]. Moreover, Gasinski et al. added mango fruit to beer in five different forms to ascertain the kind of preparation that would improve beer quality [109]. The beer prepared with fresh mango juice showed higher levels of polyphenols and antioxidant activity than those prepared with pulp or raw mango.

Along with increasing interest in the potential health benefits of beer, it is important to consider the potential negative aspects of beer consumption, which are associated with its alcohol (and energy) content. The risks of alcohol consumption are well established, with researchers having moved from the view that moderate (<14 units/week) alcohol consumption may be associated with reduced risk of mortality and, especially, of cardiovascular disease to the more contemporary view that there may be no safe threshold for alcohol consumption [178,179].

Recently, the increasing interest of consumers in health and alcohol-abuse issues focused attention of breweries on low-alcohol and non-alcoholic beers. The technical issues in producing beers with low alcohol content include the consequences of brewing to a lower ethanol content than that of regular beer, and the physical removal of ethanol after fermentation [180]. The dealcoholization approach can have undesirable effects, as it can result in cooked flavors if heat is used to evaporate the ethanol. In addition, heat might, in addition to removing alcohol, denature potential prebiotics and polyphenols. Moreover, during fermentation, yeast produces various volatile by-products, such as esters and alcohols, contributing to the aroma and flavor of the beer. The alcohol removal results in the loss of these substances. Biological methods for non-alcoholic-beer production are based on arrested fermentation, with a normal production of yeast or limited ethanol production during fermentation. Among these, the use of non-conventional yeast strains to produce low-alcohol or non-alcoholic beers, with improved flavor and aroma, is becoming popular. Non-conventional yeast strains can ferment glucose, fructose, and sucrose, but not maltose, an abundant sugar present in the wort, while producing typical concentrations of aroma compounds and generating volatile flavor compounds [181,182].

Non-alcoholic beers have been reported to exhibit phenolic contents and antioxidant activities that are somewhat lower than those of conventional beers [48,104,136,183]. The lower phenolic contents of non-alcoholic beers might be due to the limitations of the fermentation or losses during the dealcoholization procedure. Recently, the characterization of non-alcoholic beer fermented by *Pichia myanmarensis*, a non-conventional maltose-negative yeast strain, and the effects of the addition of quinoa (*Chenopodium quinoa* Wild.) have been reported [183]. The non-conventional yeast strain used in this study can ferment the simple sugars but not the maltose present in the wort [184]. The ethanol content of the beer obtained with this special yeast strain was in the non-alcoholic range (0.27–0.48% ABV). The addition of different amounts of quinoa (range, 10–30%) during the brewing process significantly increased the total polyphenol and total flavonoid contents and the antioxidant activity of the beer. Therefore, the addition of adjuncts such as fruits, vegetables, herbs, and natural foods might be used to increase the content of antioxidant bioactive molecules and the antioxidant properties of non-alcoholic beers.

## 9. Conclusions and Future Perspectives

The antioxidant properties and phenolic contents of beer rely on the quantity and quality of the starting ingredients, the adjuncts added, and the brewing process. The advent of high-resolution chromatographic techniques coupled with mass spectrometry allowed the reliable identification and accurate quantitation of phenolic molecules. The addition of adjuncts, such as fruits, vegetables, herbs, and natural foods, results in enrichment in phenolic acids, flavonoids, and stilbene molecules, thus improving the nutritional value of beer. The use of specific ingredients, such as oats, and the addition of specific adjuncts, such as olive leaves, enrich beer with particular classes of phenolic molecules, avenanthramides, and phenolic alcohol, respectively, with strong antioxidant and biological activities. In the same way, the use of wheat and rye in brewing processes may increase the alkylresorcinol concentration in the final beer. The yeast strain, the temperature, and the stage of at which the adjunct is added also influence the final composition and properties of beer. In conclusion, beer may contribute remarkably to the overall dietary intake of antioxidants, and the addition of adjuncts to beer may significantly strengthen this contribution.

Currently, there is increasing customer demand for diversity in beer styles, stimulating the search for new approaches, such as alternative yeasts and the addition of natural adjuncts to improve the taste and sensory characteristics of beers. Moreover, there is an increasing demand for low-alcohol and non-alcoholic beers. Brewing might be developed to maximize any theoretical health benefits and to minimize potential adverse effects. Therefore, careful consideration should be given to novel brewing techniques and yeasts that could help to moderate or minimize alcohol content whilst optimizing flavor, bioactive-compound contents, and healthy effects. The addition of natural adjuncts (fruits, vegetables, herbs, and natural foods) and the use of new brewing yeasts are innovative approaches to the production of special and healthy beers. Future studies need to complete the identification and characterization of the bioactive molecules in beer, as well as their absorption and metabolic fate in humans.

## Figures and Tables

**Table 2:** Bioactive compounds in conventional beers.

Phenolic Acid	mg/L Beer	Reference
Gallic acid *(free)*	0.06–10.4	[101,105,117,128,129]
Protocatechuic acid *(free)*	0.02–0.30	[101,117,128,130]
*p*-Hydroxybenzoic acid *(free)*	0.38–9.04	[117,131,132]
Gentisic acid *(free)*	0.07–0.30	[117,128]
Chlorogenic acid *(free)*	0–2.38	[115,117,130,128,129]
2,6-dihydroxybenzoic acid *(free)*	2.53 ± 0.11	[117]
Vanillic		
*free*	0–3.6	[84,101,103,104,115,116,117,128,130,132,133]
*total*	1.17–5.45	[84,103,104,115,116]
Homovanillic acid *(free)*	0.41 ± 0.04	[117]
Caffeic acid		
*free*	0–2.53	[84,103,104,105,115,116,117,128,130]
*total*	0.98–6.38	[84,103,104,115,116]
m-Hydroxybenzoic acid (*free)*	0–1.03	[117,132]
Syringic acid		
*free*	0–1.13	[84,101,103,104,115,117,128,130,133]
*total*	0–1.23	[84,103,104,115]
*p*-Coumaric acid		
*free*	0.01–5.58	[84,101,103,104,105,115,116,117,130,132]
*total*	0.55–3.10	[84,103,104,115,116]
Ferulic acid		
*free*	0.10–11.03	[84,101,103,104,115,116,117,100,128,130,131,132,133]
*total*	9.97–22.60	[84,103,104,115,116]
4-hydroxyphenylacetic acid		
*free*	0.05–1.47	[104,115,116,128,130,133]
*total*	0.40–1.46	[104,115,116]
Sinapic acid		
*free*	0.20–1.39	[84,103,104,115,116,117,130]
*total*	2.19–6.16	[84,103,104,115,116]
*m*-Coumaric acid *(free)*	0.105 ± 0.006	[117]
Salicylic acid *(free)*	0.19–6.66	[117,130]
*o*-Coumaric acid *(free)*	0.47 ± 0.04	[117]
**Flavonoids**	**mg/L beer**	**Reference**
Catechin	0.03–5.40	[101,130,129,130,131,132,133]
Epicatechin	0.02–4.55	[101,105,128,129,130,131]
Rutin	0.06–4.85	[128,129,130,131,133]
Quercetin	0.06–2.23	[128,129,132]
Kaempferol	<0.06–16.4	[129,132,133]
Daidzein	0.23–0.36	[128]
Genistein	0.06–0.08	[128]
Formononetin	0.17–1.30	[128]
Luteolin	0.10–0.19	[128]
Apigenin	0.80–0.81	[128]
Myricetin	0.15–0.16	[128]
Naringin	0.70–2.63	[128]
Naringenin	0.06–2.34	[129]
***Prenylflavonoids***	**mg/L beer**	**Reference**
8-Prenylnaringenin	0–0.021	[48,134,135,136,137,138,139,140,141]
6-Geranylnaringenin	0.001–0.074	[138]
Isoxanthohumol	0.04–3.44	[48,136,137,138]
Xanthohumol	0.002–0.69	[40,48,136,137,138]
6-Prenylnaringenin	0.011–0.56	[136,138]
**Alkylresorcinols**	**µg/L beer**	**Reference**
Total alkylresorcinols	1.01 ± 2.03	[136]
**Stilbenes**	**mg/L beer**	**Reference**
*trans*-Resveratrol	0–0.067	[142]
*cis*-Resveratrol	0–0.023	[143]
*cis*-Piceid	0–0.024	[144]
*trans*-Piceid	0–0.009	[145]
**Phenolic alcohols**	**mg/L beer**	**Reference**
Tyrosol	0.2–44.4	[117,136,146]
Hydroxytyrosol	0.0–0.13	[136,146]

Values are expressed as concentration range or as mean ± standard deviation.

**Table 3:** Antioxidant activity, and total polyphenol and flavonoid contents of beers with added fruits, vegetables, herbs, and natural foods.

Fruit Typology	Adjunct (g/L Beer)	TPC GAE mg/L ^§^	TFC CATE mg/L ^§^	FRAP mM Fe_2_SO_4_ eq. ^§^	ABTS mM TE	DPPH mM TE ^§^	Ref.
**Apples**	20	399.0 ± 11.0	67.9 ± 0.4	3.1 ± 0.07	1.62 ± 0.02	-	[103]
		(383.0–482.0)	(51.9–73.2)	(3.4–4.4)	(1.5–2.0)		
**Apricot**	200	454.0 ± 12.0	70.4 ± 0.9	4.20 ± 0.05	1.7 ± 0.04	-	[103]
		(383.0–482.0)	(51.9–73.2)	(3.4–4.4)	(1.5–2.0)		
**Cherry juice**	150-180	398.0–689.0	-	1.1–2.6 ^a^	4.8–6.5	5.2–6.4	[112]
		(315.0 ± 16.0)		(0.86 ± 0.03) ^a^	(4.63 ± 0.01)	(4.81 ± 0.23)	
**Cherry**	300	767.0 ± 1.3	221.8 ± 3.3	9.8 ± 0.11	3.5 ± 0.06	-	[103]
		(383.0–482.0)	(51.9–73.2)	(3.4–4.4)	(1.5–2.0)		
**Chestnut**	40	883.4 ± 10.9	71.7 ± 0.9	6.2 ± 0.08	3.4 ± 0.03	-	[84]
		(273.8–320.6)	(26.6–63.5)	(1.7–2.8)	(1.5–1.8)		
**Cocoa bean**	10	1026.4 ± 3.0	96.4 ± 2.0	8.1 ± 0.10	3.9 ± 0.04	-	[84]
		(382.7–446.1)	(51.9–59.0)	(3.4–3.9)	(1.5–2.6)		
**Coffee**	35	582.7 ± 6.4	69.5 ± 1.0	5.0 ± 0.14	2.9 ± 0.03	-	[84]
		(382.7–446.1)	(51.9–59.0)	(3.4–3.9)	(1.5–2.6)		
**Dotted hawthorn**	100 100	410.1 ± 11.8		1.3 ± 0.02	2.0 ± 0.12	2.2 ± 0.01	[110]
Juice		279.6 ± 2.0	-	0.9 ± 0.01	1.4 ± 0.11	0.4 ± 0.04	
Fruit		(200.5 ± 1.9)	-	(0.5 ± 0.01)	(0.9 ± 0.09)	(0.3 ± 0.03)	
**Eggplant peels extract**	10	631.0 ± 3.0	171.0 ± 9.0 ^b^	-		80.0 ± 3.17 ^c^	[108]
		(426.0 ± 12.0)	(65.0 ± 6.0) ^b^			(57.3 ± 0.37) ^c^	
**Goji berry**	50	357.0–623.0	-	-	2.4–3.8	-	[135]
		(335.0 ± 11.0)			(2.3 ± 0.11)		
**Grape**	200	631.0 ± 10.0	148.9 ± 2.0	6.8 ± 0.18	2.8 ± 0.01	-	[103]
		(383.0–482.0)	(51.9–73.2)	(3.4–4.4)	(1.5–2.0)		
**Grape**	200	501.5	-	1.3 ^a^	4.0	3.3	[158]
		(219.0)		(0.48) ^a^	(1.6)	(1.5)	
**Grape**	300	569.6 ± 4.4	-	2.6 ± 0.03 ^a^	-	1.0 ± 0.01	[159]
		(467.8 ± 6.2)		(1.3 ± 0.07) ^a^		(0.73 ± 0.03)	
**Green pepper**	6	1190.9 ± 6.7	-	-	-	78.3 ± 1.23 ^c^	[160]
		(723.2 ± 4.21)				(65.7 ± 2.0) ^c^	
**Green-pepper basil**	5	371.9 ± 1.9	-	-	-	54.9 ± 0.4 ^c^	[162]
		(291.2 ± 4.0)				(45.1 ± 0.2) ^c^	
**Green tea**	9	464.4 ± 3.9	42.0 ± 0.3	3.6 ± 0.05	2.4 ± 0.03	-	[84]
		(382.7–446.1)	(51.9–59.0)	(3.4–3.9)	(1.5–2.6)		
**Hairy-fig fruit**	100	-	-	-	-	0.41 ± 0.01	[154]
						0.12 ± 0.06	
**Hibiscus extract**	20	743.2 ± 7.0	-	-	9.28	-	[161]
		(294.2 ± 65.5)			(5.71)		
**Honey**	62	538.3 ± 8.3	48.7 ± 1.0	3.9 ± 0.01	2.5 ± 0.03	-	[84]
		(382.7–446.1)	(51.9–59.0)	(3.4–3.9)	(1.5–2.6)		
**Hop-cone extract**	0.5	316.7 ± 1.76	-	4.3 ± 0.07 ^a^	-	2.8 ± 0.03	[163]
		(280.3 ± 1.1)		(4.1 ± 0.02) ^a^		(2.5 ± 0.02)	
**Juniper-berry extract**	0.5	365.4 ± 2.8	-	4.5 ± 0.02	-	3.1 ± 0.09	[163]
		(280.3 ± 1.1)		(4.15 ± 0.02)		(2.5 ± 0.02)	
**Licorice**	2	819.7 ± 6.9	81.4 ± 1.3	6.1 ± 0.04	3.4 ± 0.01	-	[84]
		(382.7–446.1)	(51.9–59.0)	(3.4–3.9)	(1. 5–2.6)		
**Mango**	200 200 200	267.6 ± 6.9	-	1.7 ± 0.14 ^a^	1.7 ± 0.21	2.0 ± 0.09	[109]
juice		218.6 ± 4.8	-	1.3 ± 0.06 ^a^	1.2 ± 0.12	1.5 ± 0.07	
pulp		233.1 ± 6.1	-	1.5 ± 0.07 ^a^	1.3 ± 0.15	1.7 ± 0.06	
raw		(187.4 ± 6.3)	-	(1.0 ± 0.06) ^a^	(0.97 ± 0.07)	(1.4 ± 0.10)	
**Melissa extract**	0.5	363.1 ± 2.2	-	4.5 ± 0.07 ^a^	-	3.0 ± 0.08	[163]
		(280.3 ± 1.1)		(4.1 ± 0.02) ^a^		(2.5 ± 0.02)	
**Nettle-root extract**	0.5	317.2 ± 1.57	-	4.2 ± 0.04 ^a^	-	2.8 ± 0.07	[163]
		(280.3 ± 1.14)		(4.1 ± 0.02) ^a^		(2.5 ± 0.02)	
**Omija fruit**	2	606.8 ± 16.6	406.7 ± 4.0 ^b^	3.0 ± 0.05	-	2.0 ± 0.13	[111]
		(519.1 ± 15.8)	(303.2 ± 4.9) ^b^	(1.8 ± 0.09)		(0.9 ± 0.03)	
**Orange peel**	5	639.0 ± 4.0	92.4 ± 0.7	5.6 ± 0.04	2.7 ± 0.09	-	[103]
		(383–482)	(51.9–73.2)	(3.4–4.4)	(1.5–2.0)		
**Parastrephia lucida leaf**	50	800.6 ± 4.0	601.1 ± 3.0 ^b^	5.5 ± 0.04 ^a^	3.3 ± 0.11	-	[107]
		(413.2 ± 2.2)	(333.5 ± 12.8) ^b^	(1.9 ± 0.05) ^a^	(1.1 ± 0.10)		
**Peach**	50	618.4 ± 2.0	-	-	-	88.9 ± 1.3 ^c^	[157]
		(500.4 ± 4.1)				(86.1 ± 1.3) ^c^	
**Peach**	200	510.0 ± 5.0	87.3 ± 1.3	4.6 ± 0.06	1.9 ± 0.03	-	[103]
		(383–482)	(51.9–73.2)	(3.4–4.4)	(1.5–2.03)		
**Persimmon fruit**	15	701.1 ± 2.0	-	-	-	90.2 ± 1.36 ^c^	[155]
		(507.1 ± 3.0)				(80.1 ± 1.1) ^c^	
**Plum**	200	598 ± 7.0	138.8 ± 3.5	5.7 ± 0.02	1.9 ± 0.02	-	[103]
		(383–482)	(51.9–73.2)	(3.38–4.39)	(1.55–2.03)		
**Propolis extract**	0.25	306.5 ± 45.9	26.9 ± 2.7 ^b^	1.9 ± 0.25 ^a^	0.81 ± 0.20	0.6 ± 0.18	[113]
		(242.0 ± 21.2)	(16.9 ± 2.2) ^b^	(1.4 ± 0.24) ^a^	(0.63 ± 0.04)	(0.5 ± 0.16)	
**Quince**	100	159.0–175.5 ^d^	-	-	7.2–7.3	-	[156]
		(134.7 ± 8.5) ^d^			(7.1 ± 0.10)		
**Raspberry**	300	465.0 ± 6.0	90.4 ± 0.5	5.7 ± 0.09	2.3 ± 0.04	-	[103]
		(403.0 ± 5.0)	(59.0 ± 0.7)	(3.3 ± 0.03)	1.3 ± 0.02		
**Saskatoon berry**	250	377.9–413.4	-	1.6–2.0	2.18–2.22	2.4–2.9	[114]
		(243.9 ± 1.8)		(2.2 ± 0.04)	(1.8 ± 0.05)	(2.3 ± 0.07)	
**Thyme extract**	0.5	384.2 ± 3.0	-	4.7 ± 0.08 ^a^	-	3.7 ± 0.10	[163]
		(280.3 ± 1.1)		(4.1 ± 0.02) ^a^		(2.5 ± 0.02)	
**Walnut**	35	964.7 ± 9.6	90.1 ± 1.8	10.2 ± 0.02	5.2 ± 0.05	-	[84]
		(382.7–446.1)	(51.9–59.0)	(3.4–3.9)	(1.5–2.6)		

GAE: gallic-acid equivalents; CATE: catechin equivalents; TE: Trolox equivalents. Values are expressed as range of concentration or as mean ± standard deviation. Values within brackets refer to control beer without adjunct addition. ^§^ Unless otherwise specified. ^a^ FRAP values are expressed as mM TE instead of mM Fe_2_SO_4_ equivalents. ^b^ Total flavonoid contents are expressed as mM-quercetin equivalents instead of catechin equivalents. ^c^ DPPH values are expressed as percentage inhibition instead of mM TE. ^d^ Values are expressed as mg-pyrogallol equivalents instead of GAE.

**Table 4:** Bioactive phenolic compounds in beers with added fruits, vegetables, herbs, and natural foods.

Adjunct Typology	Amount Added	Bioactive Compound		Ref.
	(g/L)			
**Apple**	20	***Total phenolic acids*:** 31.2 ± 1.4 mg/L	***Flavonoids*** **:**	[103]
		*Neochlorogenic acid*: 0.79 ± 0.08 mg/L	*Catechin*: 5.2 ± 0.15 mg/L	
		*Vanillic acid*: 4.4 ± 0.07 mg/L	*Rutin*: 0.56 ± 0.01 mg/L	
		*Caffeic acid*: 4.32 ± 0.03 mg/L	*Quercetin*: 0.48 ± 0.03 mg/L	
		*Syringic acid*: 1.1 ± 0.04 mg/L	***Stilbenes:***	
		*p*-*Coumaric acid*: 2.1 ± 0.16 mg/L	*trans-Resveratrol*: 0.56 ± 0.02 mg/L	
		*Ferulic acid*: 12.7 ± 0.67 mg/L		
		*Sinapic acid*: 6.6 ± 0.40 mg/L		
**Apricot**	200	***Total phenolic acids*:** 40.7 ± 1.3 mg/L	***Flavonoids*** **:**	[103]
		*Chlorogenic acid*: 12.7 ± 0.18 mg/L	*Catechin*: 10.0 ± 0.94 mg/L	
		*Neochlorogenic acid*: 7.2 ± 0.22 mg/L	*Myricetin*: 0.99 ± 0.08 mg/L	
		*Vanillic acid*: 4.5 ± 0.06 mg/L	*Quercetin*: 3.2 ± 0.01 mg/L	
		*Caffeic acid*: 17.8 ± 0.86 mg/L	***Stilbenes*:**	
		*Syringic acid*: 0.6 ± 0.07 mg/L	*trans-Resveratrol*: 0.11 ± 0.01 mg/L	
		*p-Coumaric acid*: 2.2 ± 0.01 mg/L		
		*Ferulic acid*: 13.3 ± 0.34 mg/L		
		*Sinapic acid*: 2.2 ± 0.01 mg/L		
**Cherry juice**	150–180	***Flavonoids*:**		[112]
		*Delphinidin galactoside:* 0.1 mg/L		
		*Cyanidin galactoside*: 1.4–1.7 mg/L		
		*Cyanidin rubinoside*: 1.0–1.2 mg/L		
		*Pelargonidin galactoside*: 3.4–4.0 mg/L		
		*Pelargonidin rubinoside*: 0.9–1.0 mg/L		
		*Quercetin glucuronide*: 2.3–2.8 mg/L		
		*Kaempferol galactoside*: 1.1–1.3 mg/L		
**Cherry**	300	***Total phenolic acids*:** 145.8 ± 11.0 mg/L	***Flavonoids*** **:**	[103]
		*Chlorogenic acid*: 10.0 ± 0.36 mg/L	*Catechin*: 7.14 ± 0.10 mg/L	
		*Neochlorogenic acid*: 18.5 ± 0.6 mg/L	*Myricetin*: 1.9 ± 0.02 mg/L	
		*Vanillic acid*: 4.7 ± 0.22 mg/L	*Quercetin*: 4.0 ± 0.08 mg/L	
		*Caffeic acid*: 54.6 ± 4.3 mg/L		
		*Syringic acid*: 0.50 ± 0.05 mg/L		
		*p-Coumaric acid*: 62.4 ± 5.5 mg/L		
		*Ferulic acid*: 16.0 ± 0.34 mg/L		
		*Sinapic acid*: 7.58 ± 0.58 mg/L		
**Chestnut**	40	***Total phenolic acids*:** 45.4 ± 0.68 mg/L	***Flavonoids*** **:**	[84]
		*Vanillic acid*: 5.1 ± 0.06 mg/L	*Catechin*: 4.6 ± 0.13 mg/L	
		*Caffeic acid*: 3.5 ± 0.03 mg/L	*Epicatechin*: 3.7 ± 0.12 mg/L	
		*Syringic acid*: 1.2 ± 0.05 mg/L	***Stilbenes:***	
		*p*-*Coumaric acid*: 3.4 ± 0.07 mg/L	*trans-Resveratrol*: 0.3 ± 0.02 mg/L	
		*Ferulic acid*: 27.5 ± 0.43 mg/L		
		*Sinapic acid*: 4.7 ± 0.04 mg/L		
**Cocoa bean**	10	***Total phenolic acids*:** 38.7 ± 1.14 mg/L	***Flavonoids*** **:**	[84]
		*Vanillic acid*: 3.4 ± 0.17 mg/L	*Catechin*: 4.6 ± 0.02 mg/L	
		*Caffeic acid*: 3.7 ± 0.01 mg/L	*Epicatechin*: 1.8 ± 0.11 mg/L	
		*Syringic acid:* 1.4 ± 0.05 mg/L	*Myricetin*: 0.65 ± 0.02 mg/L	
		*p*-*Coumaric acid:* 3.3 ± 0.13 mg/L	*Quercetin*: 1.5 ± 0.06 mg/L	
		*Ferulic acid*: 22.1 ± 0.73 mg/L	***Stilbenes*:**	
		*Sinapic acid*: 4.9 ± 0.05 mg/L	*trans-Resveratrol*: 0.3 ± 0.01 mg/L	
**Coffee**	35	***Total phenolic acids*:** 36.2 ± 1.1 mg/L	***Flavonoids*** **:**	[84]
		*Chlorogenic acid*: 1.6 ± 0.10 mg/L	*Epicatechin*: 1.3 ± 0.07 mg/L	
		*Vanillic acid*: 2.0 ± 0.14 mg/L	*Myricetin*: 0.39 ± 0.03 mg/L	
		*Caffeic acid*: 9.2 ± 0.21 mg/L	*Quercetin*: 0.54 ± 0.02 mg/L	
		*p*-*Coumaric acid:* 1.9 ± 0.08 mg/L	***Stilbenes*:**	
		*Ferulic acid*: 20.5 ± 0.64 mg/L	*trans-Resveratrol:* 0.23 ± 0.01 mg/L	
		*Sinapic acid*: 2.5 ± 0.02 mg/L		
**Eggplant-peel extract**	10	***Flavonoids*** **:**		[108]
		*total monomeric anthocyanins*:		
		83.0 ± 2.0 mg/L delphinidin-3-glucoside equivalents		
**Goji berry**	50	***Phenolic acids (free forms)*:**	***Flavonoids*:**	[135]
		*p*-*Coumaric acid:* 1.4–8.0 mg/L	*Rutin*: 1.4–23.1 mg/L	
		*Ferulic acid*: 2.0–7.6 mg/L		
**Grape**	200	***Total phenolic acids*:** 49.7 ± 2.2 mg/L	***Flavonoids*** **:**	[103]
		*Chlorogenic acid*: 0.90 ± 0.04 mg/L	*Catechin*: 7.3 ± 0.59 mg/L	
		*Vanillic acid*: 7.0 ± 0.07 mg/L	*Quercetin*: 1.7 ± 0.06 mg/L	
		*Caffeic acid*: 13.4 ± 0.24 mg/L	***Stilbenes:***	
		*Syringic acid*: 2.5 ± 0.15 mg/L	*trans-Resveratrol:* 2.2 ± 0.03 mg/L	
		*p-Coumaric acid*: 7.2 ± 0.13 mg/L		
		*Ferulic acid*: 17.4 ± 1.6 mg/L		
		*Sinapic acid*: 2.2 ± 0.03 mg/L		
**Green tea**	9	***Total phenolic acids*:** 26.3 ± 1.0 mg/L	***Flavonoids*** **:**	[84]
		*Vanillic acid*: 2.8 ± 0.15 mg/L	*Catechin*: 3.0 ± 0.09 mg/L	
		*Caffeic acid*: 1.5 ± 0.18 mg/L	*Epicatechin*: 3.1 ± 0.05 mg/L	
		*Syringic acid:* 0.96 ± 0.04 mg/L	*Rutin*: 0.68 ± 0.02 mg/L	
		*p*-*Coumaric acid*: 2.2 ± 0.16 mg/L	*Myricetin*: 1.7 ± 0.05 mg/L	
		*Ferulic acid*: 14.3 ± 0.40 mg/L	*Quercetin*: 1.2 ± 0.09 mg/L	
		*Sinapic acid*: 4.5 ± 0.08 mg/L	***Stilbenes:***	
			*trans-Resveratrol*: 0.32 ± 0.02 mg/L	
**Honey**	62	***Total phenolic acids*:** 34.4 ± 0.88 mg/L	***Flavonoids*** **:**	[84]
		*Vanillic acid*: 3.1 ± 0.22 mg/L	*Epicatechin*: 0.94 ± 0.05 mg/L	
		*Caffeic acid:* 2.4 ± 0.17 mg/L	*Rutin:* 1.3 ± 0.02 mg/L	
		*Syringic acid*: 1.2 ± 0.10 mg/L	*Myricetin:* 2.7 ± 0.18 mg/L	
		*p*-*Coumaric acid*: 1.7 ± 0.03 mg/L	*Quercetin:* 4.7 ± 0.23 mg/L	
		*Ferulic acid*: 19.2 ± 0.33 mg/L	***Stilbenes*:**	
		*Sinapic acid*: 6.7 ± 0.03 mg/L	*trans-Resveratrol*: 0.24 ± 0.01 mg/L	
**Licorice**	2	***Total phenolic acids*:** 36.9 ± 1.3 mg/L	***Flavonoids*** **:**	[84]
		*Vanillic acid*: 2.3 ± 0.11 mg/L	*Rutin:* 0.92 ± 0.10 mg/L	
		*Caffeic acid*: 3.7 ± 0.04 mg/L	*Myricetin*: 8.8 ± 0.07 mg/L	
		*Syringic acid*: 0.67 ± 0.03 mg/L	*Quercetin*: 2.6 ± 0.15 mg/L	
		*p*-*Coumaric acid*: 2.9 ± 0.14 mg/L	***Stilbenes*:**	
		*Ferulic acid*: 20.6 ± 0.87 mg/L	*trans-Resveratrol*: 0.20 ± 0.01 mg/L	
		*Sinapic acid*: 6.7 ± 0.07 mg/L		
**Olive leaf**	9.9	***Oleuropein*:** 42–73 mg/L		[164]
		***3-hydroxytyrosol*:** 43–75 mg/L		
**Omija fruit**	2	***Lignans*:**		[111]
		*Schisandrin*: 9.0–12.1 mg/L		
		*Gomisin A*: 2.2–3.1 mg/L		
		*Gomisin B*: 0.65–0.86 mg/L		
**Orange peel**	5	***Total phenolic acids*:** 48.1 ± 1.7 mg/L	***Flavonoids*** **:**	[103]
		*Vanillic acid*: 4.6 ± 0.45 mg/L	*Catechin*: 10.9 ± 0.70 mg/L	
		*Caffeic acid*: 3.6 ± 0.09 mg/L	*Rutin:* 1.5 ± 0.17 mg/L	
		*Syringic acid*: 0.55 ± 0.02 mg/L	*Myricetin*: 0.76 ± 0.04 mg/L	
		*p*-*Coumaric acid*: 4.9 ± 0.13 mg/L	*Quercetin*: 0.76 ± 0.01 mg/L	
		*Ferulic acid*: 27.9 ± 0.48 mg/L		
		*Sinapic acid*: 6.5 ± 0.55 mg/L		
**Peach**	200	***Total phenolic acids*:** 35.8 ± 2.0 mg/L	***Flavonoids*** **:**	[103]
		*Chlorogenic acid*: 0.81 ± 0.05 mg/L	*Catechin*: 5.7 ± 0.10 mg/L.	
		*Neochlorogenic acid*: 3.4 ± 0.11 mg/L	*Myricetin:* 1.8 ± 0.07 mg/L.	
		*Vanillic acid*: 6.1 ± 0.76 mg/L	*Quercetin*: 0.30 ± 0.01 mg/L.	
		*Caffeic acid*: 16.3 ± 0.87 mg/L	***Stilbenes:***	
		*Syringic acid*: 0.72 ± 0.02 mg/L	*trans-Resveratrol*: 1.0 ± 0.04 mg/L.	
		*p*-*Coumaric acid*: 1.3 ± 0.03 mg/L		
		*Ferulic acid*: 9.2 ± 0.23 mg/L		
		*Sinapic acid*: 2.2 ± 0.10 mg/L		
**Plum**	200	***Total phenolic acids*:** 119.8 ± 8.7 mg/L	***Flavonoids*** **:**	[103]
		*Chlorogenic acid*: 8.94 ± 0.22 mg/L	*Catechin*: 6.4 ± 0.37 mg/L	
		*Neochlorogenic acid*: 60.3 ± 1.2 mg/L	*Myricetin*: 5.3 ± 0.32 mg/L	
		*Vanillic acid*: 2.7 ± 0.31 mg/L	*Quercetin*: 1.5 ± 0.06 mg/L	
		*Caffeic acid*: 89.8 ± 7.5 mg/L		
		*Syringic acid:* 0.58 ± 0.03 mg/L		
		*p*-*Coumaric acid*: 12.2 ± 0.58 mg/L		
		*Ferulic acid:* 14.2 ± 0.29 mg/L		
		*Sinapic acid*: 0.33 ± 0.02 mg/L		
**Quince**	100	***Total hydroxycinnamic acids*:**		[156]
		32.4–35.6 mg/L		
		*Neochlorogenic acid*: 3.9–5.1 mg/L		
		*Chlorogenic acid*: 6.5–9.7 mg/L		
		*3,5-Dicaffeoylquinic acid*: 3.0–3.2 mg/L		
**Raspberry**	300	***Total phenolic acids*:** 26.5 ± 0.80 mg/L	***Flavonoids*** **:**	[103]
		*Chlorogenic acid*: 0.84 ± 0.10 mg/L	*Catechin*: 6.0 ± 0.49 mg/L	
		*Neochlorogenic acid*: 2.6 ± 0.22 mg/L	*Myricetin*: 1.5 ± 0.10 mg/L	
		*Vanillic acid*: 5.1 ± 0.22 mg/L	*Quercetin*: 3.0 ± 0.12 mg/L	
		*Caffeic acid*: 2.8 ± 0.20 mg/L	***Stilbenes:***	
		*Syringic acid*: 1.2 ± 0.09 mg/L	*trans-Resveratrol:* 0.14 ± 0.01mg/L	
		*p*-*Coumaric acid*: 1.2 ± 0.12 mg/L		
		*Ferulic acid*: 13.1 ± 0.08 mg/L		
		*Sinapic acid*: 3.1 ± 0.08 mg/L		
**Saskatoon berry**	250	***Phenolic acids (free)*** **:**	***Flavonoids*** **:**	[114]
		*Caffeic acid*: 0.87–0.96 mg/L	*Kaempferol-3-O-glc-pent:* 0.66–0.80 mg/L	
		*Chlorogenic acid*: 1.46–2.17 mg/L	*Kaempferol-3-O-rut:* 0.78–0.81 mg/L	
		*Neochlorogenic acid:* 1.07–1.21 mg/L	*Kaempferol-3-O-rha-7-O-pent:* 0.94–0.97 mg/L	
		*Sinapic acid glucoside*: 1.05–2.23 mg/L		
		*Ferulic acid derivatives*: 0.79–1.00 mg/L		
**Walnut**	35	***Total phenolic acids*:** 20.5 ± 0.88 mg/L	***Flavonoids*** **:**	[84]
		*Vanillic acid*: 2.2 ± 0.26 mg/L	*Epicatechin*: 1.8 ± 0.11 mg/L	
		*Caffeic acid*: 3.2 ± 0.15 mg/L	*Myricetin*: 4.4 ± 0.27 mg/L	
		*p*-*Coumaric acid*: 4.3 ± 0.24 mg/L	*Quercetin:* 6.5 ± 0.31 mg/L	
		*Ferulic acid*: 8.2 ± 0.17 mg/L	***Stilbenes:***	
		*Sinapic acid*: 2.7 ± 0.06 mg/L	*trans-Resveratrol*: 0.26 ± 0.20 mg/L	

Values represent concentration range or mean ± standard deviation. Glc, glucoside; pent, pentoside; rut, rutinoside; rha, rhamnoside.

## Data Availability

The data presented in this study are available on request from the corresponding author.

## Abbreviations

CAS, Chemical Abstract Service; DW, dry weight; FW, fresh weight; GAE, gallic-acid equivalents; TE, Trolox equivalents; ARs, alkylresorcinols; glc, glucoside; pent, pentoside; rut, rutinoside; rha, rhamnoside; ABTS, 2,2′-azino-bis(3-ethylbenzothiazoline-6-sulfonic acid assay; FRAP, ferric reducing antioxidant power assay; DPPH, 2,2-diphenyl-1-picrylhydrazyl assay; LC-MS, liquid chromatography–mass spectrometry; HPLC, high-performance liquid chromatography; HPLC-MS; high-performance liquid chromatography–mass spectrometry; UHPLC, ultra-high-performance liquid chromatography; UHPLC-MS, ultra-high-performance liquid chromatography–mass spectrometry; GC, gas chromatography; GC-MS, gas chromatography–mass spectrometry; NMR, nuclear magnetic resonance; HPLC-ESI-MS/MS, high-performance liquid chromatography–electrospray ionization–mass spectrometry; LC-Aox, liquid chromatography–antioxidant; ABV, alcohol by volume.
